# YWHAE as an HE4 interacting protein can influence the malignant behaviour of ovarian cancer by regulating the PI3K/AKT and MAPK pathways

**DOI:** 10.1186/s12935-021-01989-7

**Published:** 2021-06-09

**Authors:** Xiao Li, Caixia Wang, Shuang Wang, Yuexin Hu, Shan Jin, Ouxuan Liu, Rui Gou, Xin Nie, Juanjuan Liu, Bei Lin

**Affiliations:** Department of Obstetrics and Gynecology, Shengjing Hospital Affiliated to China MedicalUniversity, No. 36, Sanhao Street, Heping District, Shenyang, 110004 People’s Republic of China

**Keywords:** YWHAE, HE4, Interacting protein, Poor prognosis, Malignant behaviour, Ovarian cancer

## Abstract

**Background:**

Malignant tumours of the female reproductive system threaten the lives and health of women worldwide, with ovarian cancer having the highest mortality rate. Based on previous work, this study analysed the expression and role of YWHAE in ovarian epithelial tumours.

**Methods:**

The interaction between YWHAE and HE4 was evaluated via immunoprecipitation, western blot analysis, and cellular immunofluorescence. Immunohistochemistry was used to address the relationship between YWHAE expression, clinicopathological parameters, and patient prognosis. Changes in cell invasion, epithelial–mesenchymal transition, migration, proliferation, apoptosis, and cell cycle before and after differential expression of YWHAE were also explored in ovarian cancer cell lines and via in vivo experiments.

**Results:**

YWHAE was found to interact with HE4, and its expression was positively correlated with HE4 expression. Moreover, YWHAE upregulation was associated with advanced stages of ovarian cancer and poor patient prognosis. In addition, YWHAE enhanced invasion, migration, and proliferation, but inhibited the apoptosis of ovarian cancer cells. These biological effects were found to be mediated by the AKT and MAPK signalling pathways.

**Conclusions:**

Altogether, this study demonstrates that YWHAE is substantially upregulated in ovarian cancer tissues, representing a risk factor for the prognosis of ovarian cancer that is positively correlated with HE4 expression. Furthermore, YWHAE and its downstream pathways may represent new therapeutic targets for ovarian cancer.

**Supplementary Information:**

The online version contains supplementary material available at 10.1186/s12935-021-01989-7.

## Introduction

Ovarian cancer is among the most common malignant tumours of the female reproductive system and has the highest mortality rate. Due to the lack of obvious or specific symptoms, or reliable screening methods, rates of early detection and diagnosis of ovarian cancer are extremely low [1], resulting in advanced tumor progression, and shortened tumour-free and overall survival times [2]. Therefore, there is an urgent need to identify tumour markers with high sensitivity and specificity to guide early clinical screening and diagnosis and for monitoring ovarian cancer.

Human epididymis protein 4 (HE4) is a highly sensitive and specific ovarian cancer marker that has been identified via genomics and proteomics screenings [3]. Researchers have shown that HE4 detection is more advantageous than the detection of the common tumour marker CA125 for the early diagnosis of ovarian cancer and for monitoring disease progression [4]. In 2003, HE4 was designated as a serum marker for ovarian cancer and was approved by the U.S. Food and Drug Administration in 2009 for monitoring the recurrence and progression of epithelial ovarian cancer [5]. Currently, several ongoing clinical studies are addressing the diagnostic potential of HE4; however, few are exploring its underlying molecular mechanisms.

Our previous study has demonstrated that HE4 is highly expressed in ovarian cancer tissues, and that the interaction between HE4 and annexin A2 promotes the invasion and metastasis of ovarian cancer. This is accomplished by activating adhesion signalling pathways, such as mitogen-activated protein kinase (MAPK) and FOCAL [6, 7]. Proteins perform their biological functions primarily by forming protein complexes, or by working with chaperone molecules. The coordination of proteins with different functions constitutes the basic process of life. Therefore, given the crucial role of HE4 in ovarian cancer, it is clear that recognition and improved knowledge on HE4-interacting proteins is an important step for a better understanding of this disease. A previous study using HE4 as bait in a two-hybrid screen identified the tyrosine 3-monooxygenase/tryptophan 5-monooxygenase activation protein epsilon (YWHAE) protein as a partner for HE4.

YWHAE, also known as 14-3-3ε, belongs to the YWHA protein family, which comprises at least seven highly conserved subtypes of soluble acidic proteins encoded by different genes, namely β, ε, η, γ, τ, ξ, and σ [8]. It can be widely combined with other proteins such as kinases, phosphatases, transmembrane receptors, and transcription factors, among other target proteins [9–11], to function as a protein interaction bridge in a wide range of biological processes [12–14].

X-ray diffraction analysis showed that YWHAE protein monomers form homodimers or heterodimers [15] that are bound by highly conserved hydrophobic amino acids. Different YWHA subtypes can bind to the same target, giving these proteins the ability to regulate many physiological processes, including cell proliferation, apoptosis, protein transport, metabolic regulation, and signal transduction, among others [16, 17]. The structure of YWHAE may be the basis for its role as a “bridge protein”, as well as its contribution towards disease incidence [18].

Based on previous preliminary findings, this study explored the relationship between YWHAE and HE4 and analysed the expression of YWHAE in ovarian epithelial tumours, as well as its mechanism of action. Improved knowledge regarding the activity of YWHAE and HE4 may provide a basis to further explore the development and progression of ovarian cancer and to design novel diagnostic strategies.

## Materials and methods

### Primary samples

Ovarian tissue samples were surgically collected between 2008 and 2012 from patients at the Department of Obstetrics and Gynecology, Shengjing Hospital, China Medical University. A total of 16 samples were derived from normal ovarian tissue removed due to uterine fibroids or cervical cancer (normal group), 18 samples were from benign cases, 24 samples were classified as borderline, and 105 samples were from malignant ovarian cancers. The pathological types among the malignant samples included 71 serous tumours, seven mucinous tumours, 19 endometrioid tumours, and eight clear cell carcinomas. The malignant group was also classified according to pathology assessment, with 51 cases identified as highly differentiated and 54 as poorly differentiated. Surgical pathological staging was performed in accordance with the International Federation of Obstetrics and Gynecology (FIGO) standards: 44 cases were in stage I–II and 61 were in stage III–IV. A comprehensive exploratory surgery was performed in stage I–II, and cytoreductive surgery was performed in stage III–IV. In the malignant group, 92 patients underwent lymph node dissection, with lymph node metastasis being confirmed in 28 cases. All cases were newly diagnosed, and patients were radiotherapy- and chemotherapy-naïve.

### Immunochemistry

Histopathological specimens were fixed with 10% formalin solution, embedded in paraffin, and then serially sectioned into 5 μm slices. The paraffin sections were deparaffinised with xylene and re-hydrated with gradient alcohol solutions, and the antigens were recovered by heating. Subsequently, H_2_O_2_, goat serum blocking solution, and anti-YWHAE antibody (1:100, sc-23957, Santa Cruz Biotechnology, Santa Cruz, CA) or an anti-HE4 antibody (1:1500, ab200828, Abcam, Cambridge, UK) were sequentially added in a dropwise manner; the solutions were left to incubate overnight at 4 °C. The following day, the slices were incubated with horseradish peroxidase (HRP)-labelled goat anti-rabbit/mouse secondary antibodies and stained using 3,3-diaminobenzidine (UltraSensitive™ SP Mouse/Rabbit IHC Kit, Fuzhou Maixin Biotech Co. Ltd., Fuzhou, China). Nuclei were stained blue using haematoxylin. The sections were then dehydrated, cleared by xylene, and mounted.

Samples were independently observed, scored, and evaluated by two pathologists who were blinded to the clinical information of the patients. If discordant scoring results were obtained, a third pathologist would assess the sample for the final decision. The samples were classified as positive when presenting with a brownish-yellow, or brown colour in the cell cytoplasm and/or membrane. If the proportion of positive cells was less than 5%, it was scored as 0 points, 5–25% was scored as 1 point, 26–50% was 2 points, 51–75% was 3 points, and more than 75% was scored as 4 points. According to the colour intensity, samples were further scored with 3 points for brown, 2 points for brownish-yellow, 1 point for light yellow, and 0 points for no staining. To reach a final score, the two classifications were multiplied: 0–2 points was recorded as negative expression (−), 3–4 points as weakly positive expression (+), 5–8 points as moderately positive expression (++), and 9–12 points as strongly positive expression (+++).

### Cellular immunofluorescence

Cells were seeded on a microscope slide and washed with phosphate-buffered saline (PBS) after they had adhered to the glass. Goat serum blocking solution was added, followed by the anti-YWHAE (1:100, sc-23957, Santa Cruz Biotechnology, Santa Cruz, CA) and anti-HE4 (1:200, DF8160, Affinity Biosciences, Cincinnati, OH) primary antibody mix. A secondary antibody mixture containing tetramethylrhodamine-labelled goat anti-rabbit IgG (SA00007-2, Proteintech Group Inc., Wuhan, China) and fluorescein isothiocyanate-labelled goat anti-mouse IgG (SA00003-1, Proteintech Group Inc., Wuhan, China) was dropped onto the slide, which was then incubated for 2 h in the dark. Nuclei were stained with 4′,6-diamidino-2-phenylindole (4083S; Cell Signaling Technology Inc., Danvers, MA), and an anti-quenching agent was added dropwise onto the slide immediately before viewing the slices on a confocal microscope.

### Establishment of stable cell lines overexpressing YWHAE and transient YWHAE knockdown cell lines

The ovarian cancer cell lines, CAOV3 and ES2 (Shanghai Institute of Biochemistry and Cell Biology, Chinese Academy of Sciences, Shanghai, China), were cultured in RPMI 1640 Medium (Biological Industries, Beit-Haemek, Israel) supplemented with 10% foetal bovine serum (Biological Industries, Beit-Haemek, Israel). When the cells reached 80–90% confluence, the medium was discarded, the cells were washed with PBS, trypsinized, and split to continue the culture. *YWHAE-*siRNA (5′-GAAGCAGGUUAGCGUUGAATT-3′ and 3′-UUCAACGCUAACCUGCUUCTT-5′), *Mock-YWHAE-*siRNA (5′-GAAGCAGGUUAGCGUUAGATTUU-3′ and 3′-UUCTTCGCUAACCCTCUUCTT-5′) (Additional file 1), *HE4-*siRNA (5′-AGGUGAACAUUAACUUUCCTT-3′ and 3′-GGAAAGUUAAUGUUCACCUTT-5′), and *Mock-HE4*-siRNA (5′-AGGUGAACACCAACTTUCCTT-3′ and 3′-GGAAAGCCAAUGUUTCCCUTT-5′) working solutions were prepared according to the manufacturer’s instructions (GenePharma, Suzhou, China). CAOV3 and ES2 cells were seeded into 6-well plates with serum-free medium on the day of transfection. *YWHAE-siRNA* and *HE4-siRNA* were transfected into these cell lines using Lipofectamine 3000 (Invitrogen, Waltham, MA, USA) according to the manufacturer’s instructions.

A lentivirus-mediated *YWHAE* overexpression vector was used to transduce OVCAR3 and A2780 cell lines, which have relatively low YWHAE expression. To determine the multiplicity of infection of the YWHAE-expressing lentivirus, 500 µL of complete medium was added to a 24-well plate, in addition to lentivirus supernatant and a corresponding volume of polybrene to promote transduction. Puromycin at 50% lethal concentration was used to select the transduced cells.

### Western blotting

Total protein samples were separated using sodium dodecyl sulphate polyacrylamide gel electrophoresis. Briefly, 5–10 µL of each protein sample was electrophoresed at 80–120 V under a constant electrical current for 40–100 min. The proteins were then transferred onto a polyvinylidene fluoride membrane (Millipore, Burlington, MA), and blocked with a 5% milk/bovine serum albumin solution for 2 h at 37 °C. The membrane was incubated overnight at 4 °C with primary antibodies (YWHAE, 1:1000, ab92311, Abcam, Cambridge, UK; HE4, 1:1500, DF8160, Affinity Biosciences, Cincinnati, OH; cyclin D1, 1:1000, 2978S, Cell Signaling Technology Inc., Danvers, MA; Ki-67, 1:1000, 9449S, Cell Signaling Technology Inc., Danvers, MA; Bax, 1:1000, 5023S, Cell Signaling Technology Inc., Danvers, MA; Bcl-2, 1:1000, 12789-1-AP, Proteintech Group Inc., Wuhan, China; MMP2, 1:1000, 10373-2-AP, Proteintech Group Inc., Wuhan, China; MMP9, 1:1000, 10375-2-AP, Proteintech Group Inc., Wuhan, China; E-cadherin, 1:1000, 20874-1-AP, Proteintech Group Inc., Wuhan, China; N-cadherin, 1:1000, 4061S, Cell Signaling Technology Inc., Danvers, MA; Vimentin, 1:3000, 10366-1-AP, Proteintech Group Inc., Wuhan, China; PI3K, 1:1000, 4292S, Cell Signaling Technology Inc., Danvers, MA; p-PI3K, 1:1000, 4228S, Cell Signaling Technology Inc., Danvers, MA; AKT, 1:1000, 4691S, Cell Signaling Technology Inc., Danvers, MA; p-AKT, 1:1000, 4060S, Cell Signaling Technology, Beverly, MA; m-TOR, 1:1000, 2972S, Cell Signaling Technology Inc., Danvers, MA; p-m-TOR, 1:1000, 2971S, Cell Signaling Technology Inc., Danvers, MA; MEK, 1:1000, sc-81504, Santa Cruz Biotechnology, Santa Cruz, CA; p-MEK, 1:1000, 2338S, Cell Signaling Technology Inc., Danvers, MA; ERK, 1:1000, 9102S, Cell Signaling Technology Inc., Danvers, MA; and p-ERK, 1:1000, 9101S, Cell Signaling Technology Inc., Danvers, MA). After washing the membrane with PBS-Tween, the membrane was incubated with HRP-labelled goat anti-rabbit (1:3000, ZB-2301, ZSGB-BIO, Beijing, China) or goat anti-mouse (1:3000, ZB-2305, ZSGB-BIO, Beijing, China) secondary antibodies for 1 h at 37 °C, and then washed again. Finally, western chemiluminescent HRP Substrate (Thermo Fisher Scientific, Waltham, MA) was added dropwise onto the membrane, and luminescent signals were detected on a luminometer at different exposure times.

### Immunoprecipitation

Cells in the exponential growth phase were collected and washed. Ice-cold lysis buffer was added, and the cell suspension was sonicated. The supernatant was collected and the total protein concentration was determined. Samples (500 μg) were then incubated with 2 μg of YWHAE (1:100, sc-23957, Santa Cruz Biotechnology, Santa Cruz, CA) or HE4 (1:1500, ab200828, Abcam, Cambridge, UK) primary antibodies. An IgG antibody (5145S, Cell Signaling Technology Inc., Danvers, MA) of the same species as the primary antibodies was used as a negative control. Following mixing, the samples were rotated slowly overnight at 4 °C. Subsequently, Protein A/G Plus-Agarose Beads (sc-2003, Santa Cruz Biotechnology, Santa Cruz, CA) were added to each tube and incubated for 6 h. Bound proteins were collected via centrifugation, (2×) loading buffer was added, and the samples were boiled to denature the proteins.

### Invasion test

A transwell insert was placed into a 24-well plate. Matrigel (1:7.5 dilution, 356234, BD Biosciences, Franklin Lakes, NJ) and serum-free cell suspensions (4 × 10 cells) were added to the upper insert of the chamber, whereas the lower chamber contained complete medium (with 10% serum). After culturing for 48–72 h, the cells in the lower chamber were collected, fixed, and stained. Residual cells left on plate were observed under a microscope. The experiment was repeated thrice.

### Scratch test

Cells were seeded in a 6-well culture plate and maintained at 37 °C in an atmosphere of 5% CO_2_. When the cells were at 90% confluence, the monolayer was scratched with a 100 µL pipette tip. Cells were cultured in serum-free medium for 24 h, and then washed with PBS and imaged to monitor the healing of the scratch. The experiment was repeated thrice.

### Cell viability assay

A total of 2 × 10^3^ cells/well were seeded in to a 96-well culture plate and maintained at 37 °C in an atmosphere of 5% CO_2_. After the cells had adhered, 20 µL of sterile MTT (M8180,Solarbio, Beijing, China) working solution was added to each well, mixed well, and the cells incubated at 37 °C for 4 h. Following this, the medium was aspirated and 150 µL of DMSO (D8370, Beijing Solarbio Science & Technology Co Ltd., Beijing, China) was added to each well. The absorbance of each well was measured using a microplate reader after shaking for 5 min. The experiment was repeated thrice.

### Cell cycle analysis

Cells in the log phase of replication were collected and washed, and pre-cooled ethanol was slowly added to fix the cells for later use. Prior to analysis, a total of 500 µL PI/RNase A (KGA512, Nanjing KeyGen Biotech Co Ltd., Nanjing, China) staining solution was added to the cell suspension, and the cells were incubated in the dark for 20 min, according to the manufacturer’s instructions. The cells were analysed via flow cytometry. The experiment was repeated thrice.

### Apoptosis assessment

The annexin V/PI double staining method was used to evaluate the impact of YWHAE overexpression on ovarian cancer cells. The cells were trypsinized without EDTA, centrifuged, and the supernatant was discarded. Cells were then slowly resuspended in 500 µL of Binding Buffer and incubated with 5 µL of annexin V-APC (550474, BD Biosciences, Franklin Lakes, NJ)/FITC (KGA107, Nanjing KeyGen Biotech Co Ltd., Nanjing, China) and PI in the dark at 37 °C for 15 min, according to the manufacturer’s instructions. Apoptosis was evaluated via flow cytometry.

### In vivo xenograft model of ovarian cancer

Twenty female nude mice (Huafukang Biosciences, Beijing, China) were randomly divided into two groups and injected with either OVCAR3-YWHAE-MOCK or OVCAR3-YWHAE-H ovarian cancer cells. Approximately 100 µL of cell suspension containing 1 × 10^7^ cells was injected subcutaneously into the armpit of the right forelimb of each mouse. Tumour progression and overall health status of the mice were observed every 3 days, and the diameter of the tumour and the weight of the mice were measured. The tumour volume was calculated using the following formula: V = (a × b^2^)/2, where (a) represents the largest diameter, and (b) the shortest diameter. On day 7 post-injection, newly formed tumours were detectable; on day 21, the largest tumour was nearly 1 cm in diameter, and the mice had started to show poor health symptoms. The tumour samples were then fixed in 4% paraformaldehyde and embedded in paraffin. Continuous 5 μm-thick sections were cut and analysed using either haematoxylin and eosin or immunohistochemical staining. This animal study was approved by the Institutional Animal Research Committee of China Medical University.

### Signalling pathway inhibition

YWHAE-overexpressed and mock-transduced cells were cultured in the presence of 25 µmol/L of the PI3K inhibitor (GDC-0941, Selleck Chemicals, Houston, TX) or 20 µmol/L of a selective inhibitor of mitogen-activated protein kinase 1 and 2 (MAPK1/2) (PD98059, Selleck Chemicals, Houston, TX). DMSO (0.1%) served as the negative control.

### Bioinformation analysis

The YWHAE co-expression gene set was downloaded from cBioPortal (www.cbioportal.org), and the top 300 genes were annotated according to the Gene Ontology (GO) (BP, biological process; CC, cellular component; and MF, molecular function) and the Kyoto Encyclopaedia of Genes and Genomes (KEGG) pathway analysis databases, using the tools available in DAVID (https://david.ncifcrf.gov/).

The Oncomine database (http://www.oncomine.org), which is currently the world’s largest oncogene chip database integrated with a data mining platform, was used to analyse the mRNA expression level of YWHAE in the different cancer cell types.

The interaction network among the top 100 genes was constructed using STRING (www.string-db.org) and Cytoscape (www.cytoscape.org). The protein–protein interaction (PPI) network was constructed using the STRING (http://string.embl.de/) online tool. The visualization plot was generated using Cytoscape software with a confidence score of ≥ 0.1 defined as the cut-off. The core modules of the PPI network were screened using MCODE with the following parameters: degree threshold = 2, node threshold = 0.2, kcore = 2, and maximum depth = 100.

### Statistical analysis

Statistical differences between two groups were evaluated using a Student’s *t* test, and one-way analysis of variance (ANOVA) was used for comparison of more than two groups. The data were counted using the χ^2^ and Fisher’s exact probability tests. *P* < 0.05 was considered statistically significant.

## Results

### YWHAE and HE4 are interacting proteins in ovarian cancer

**Fig. 1:**
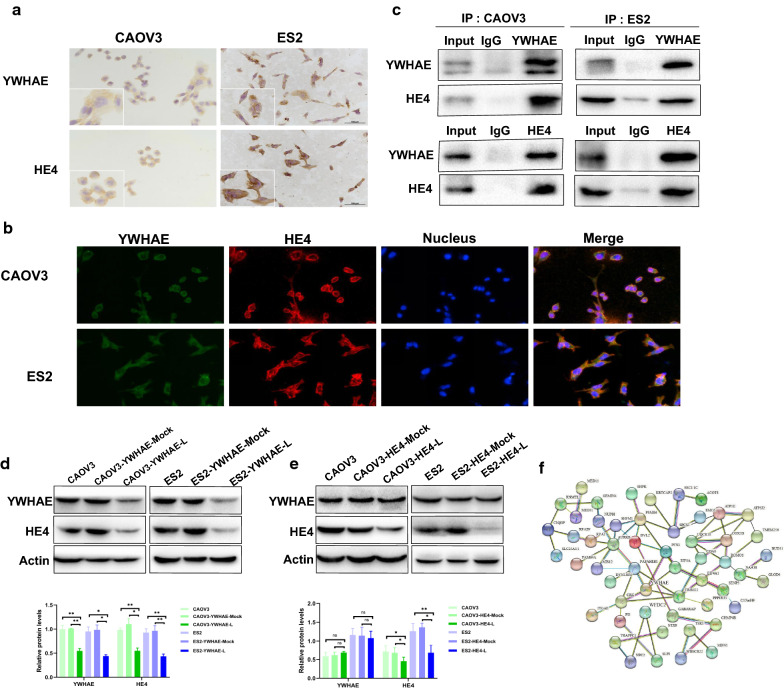
YWHAE and HE4 are interacting proteins in ovarian cancer. **a** Expression of YWHAE and HE4 in CAOV3 and ES2 cells. **b** Immunofluorescence assay to detect the co-localisation of YWHAE and HE4. **c** Co-IP detection of interactions between YWHAE and HE4 in CAOV3 (left) and ES2 (right) cell lines. The first channel shows the Co-IP, the second shows IgG, and the third channel shows total protein. **d**, **e** Western blotting was used to detect the relationship between YWHAE and HE4. **f** Bioinformatics analysis showed that HE4 acts as an interaction factor for YWHAE

The levels of YWHAE and HE4 were evaluated in CAOV3 and ES2 ovarian cancer cell lines using cellular immunohistochemistry, revealing that both proteins were expressed in these lines (Fig. 1a), co-localising in the cytoplasm and cell membranes (Fig. 1b). Co-immunoprecipitation of YWHAE and HE4 in these cell lines further demonstrated that they are interacting proteins (Fig. 1c). Additionally, siRNA-mediated knockdown of *YWHAE* led to reduced levels of HE4 in both CAOV3 and ES2 transiently transfected cells, whereas *HE4* knockdown had no significant effect on YWHAE levels. These results suggest that YWHAE works upstream of HE4 (Fig. 1d,e), and can regulate HE4 expression.

DAVID analysis of the top 300 genes identified on the GO (BP/CC/MF) and KEGG databases showed an enrichment of 36 BP, which included RNA scattering, Wnt signalling, and cell adhesion, 23 CC, and 13 MF (all *P* < 0.05). In addition, two KEGG signalling pathways were identified (all *P* < 0.05), which included spliceosome and RNA transport. A bubble diagram of the top five GO (BP/CC/MF) and KEGG pathways showed that YWHAE may interact with PAFAH1B1, EIF5A, PITPNA, TIMM22, PFN1, and WFDC2 (which is another name of HE4) (Fig. 1f).

### Expression and correlation analysis of YWHAE and HE4 in each group of ovarian tissues

#### YWHAE expression in ovarian tissues

**Fig. 2:**
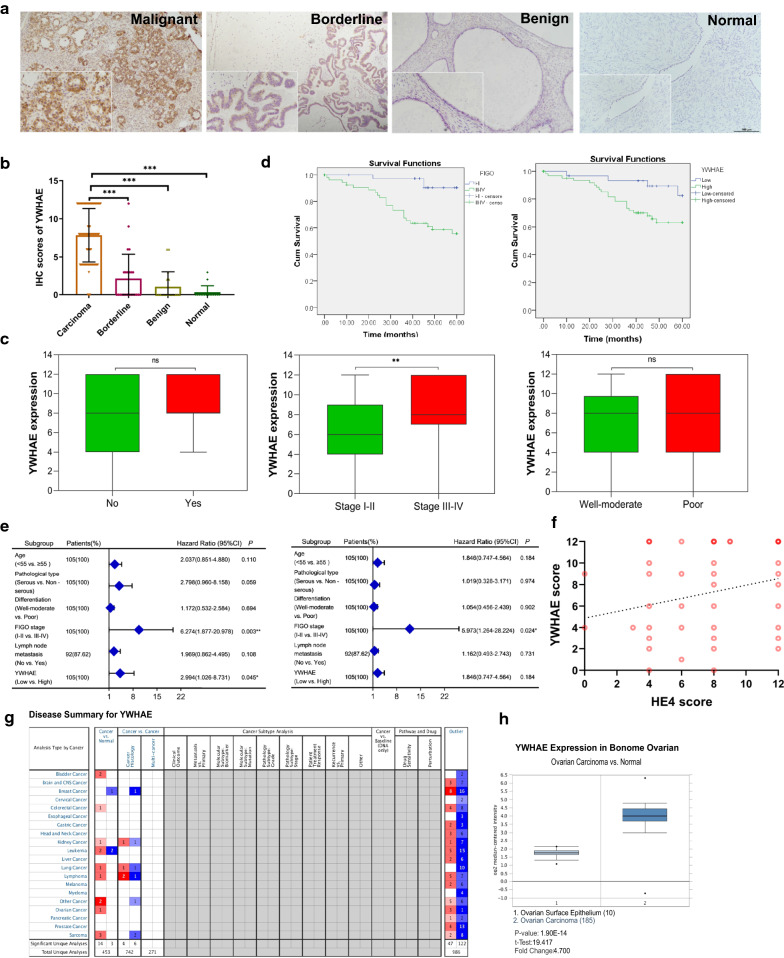
YWHAE expression in clinical specimens. **a** The expression of YWHAE and HE4 in the same position in malignant, borderline, benign, and normal tissues. **b** YWHAE scores in different ovarian tissues. **c** Comparison of YWHAE expression between groups with and without lymph node metastasis, FIGO stage I–II vs. III–IV, and groups with well-moderate or poor differentiation. **d** The influence of FIGO stage and YWHAE expression on the survival and prognosis of ovarian cancer patients. **e** Univariate and multivariate Cox analyses of different clinicopathological parameters of ovarian cancer. **f** Linear correlation analysis between YWHAE and HE4 expression in ovarian cancer; **g**, **h** YWHAE expression in Oncomine database

Consistent with the results from cell line studies, analysis of primary tissue samples showed that YWHAE was mainly located in the cytoplasm and cell membrane. Approximately 96.19% (101/105) of the malignant samples were positive for YWHAE with a strongly positive rate of 70.48% (74/105), whereas the borderline group had a YWHAE-positive rate of 41.67% (10/24), and a strongly positive rate of 16.67% (4/24). Benign and normal ovarian tissue samples had a positive rate of 16.67% (3/18) and 6.25% (1/16), and a strongly positive rate of 11.11% (2/18) and 0.00% (0/16), respectively. Comparison between the different groups revealed that YWHAE-positive expression was significantly higher in the malignant group (*P* < 0.05); however, the borderline group also had a higher YWHAE-positive rate than that of the benign and normal groups (*P* < 0.05). The expression rate of YWHAE in the benign group was higher than in the normal group, however the difference was not statistically significant (*P* > 0.05) (Table 1, Fig. 2a, b).

**Table 1:** Expression of YWHAE in different ovarian tissues

Groups	Cases	Low		High		Positive rate (%)	High expression rate (%)
		−	+	++	+++		
Malignant	105	4	27	36	38	96.19	70.48
Borderline	24	14	6	2	2	41.67	16.67
Benign	18	15	1	2	0	16.67	11.11
Normal	16	15	1	0	0	6.25	0.00

#### Relationship between YWHAE expression and the clinicopathological parameters of ovarian cancer

To compare clinicopathological parameters with the expression of YWHAE in ovarian tissue, we reviewed clinical information of the 105 patients with primary malignant epithelial ovarian tumours. Analysis of the pathological data showed that a strongly positive YWHAE expression rate was significantly higher in FIGO stage III–IV ovarian epithelial malignancies than in the early-stage group (80.33% versus 56.82%, *P* < 0.01). No statistical differences were observed in any other clinicopathological parameters (Table 2, Fig. 2c).

**Table 2:** Relationships between the expression of YWHAE and clinicopathological parameters

Groups	Cases	Low		High		Positive rate (%)	*P-*value	High expression rate (%)	*P-*value
		(−)	(+)	(++)	(+++)				
Age at diagnosis									
< 55	54	4	13	18	19	92.59	*P* = 0.066	68.52	*P* = 0.651
≥ 55	51	0	14	18	19	100.00		72.55	
Pathological type									
Serous	71	2	14	28	27	97.18	*P* = 0.222	77.46	*P* = 0.073
Mucinous	7	1	3	2	1	85.71		42.86	
Endometrioid	19	1	6	4	8	94.73		63.16	
Clear cell carcinoma	8	0	4	2	2	100.00		50.00	
FIGO stage									
I–II	44	3	16	12	13	93.18	*P* = 0.307	56.82	*P* = 0.009
III–IV	61	1	11	24	25	98.36		80.33	
Differentiation									
Well and moderate	51	3	13	12	23	94.12	*P* = 0.354	68.63	*P* = 0.687
Poor	54	1	14	24	15	98.15		72.22	
Lymphatic metastasis									
No	64	4	18	22	20	93.75	*P* = 0.310	65.63	*P* = 0.214
Yes	28	0	6	10	12	100.00		78.57	
Unknown^a^	13	0	3	4	6	100.00		76.92	

^a^13 patients without lymphadenectomy

#### Relationship between YWHAE expression and the survival of patients with ovarian cancer

A follow-up of these patients further revealed that only four deaths occurred in the low YWHAE expression group (n = 31), while 21 deaths were recorded in the high YWHAE expression group (n = 74). Kaplan–Meier survival analysis showed that the survival rate among patients with high YWHAE expression was significantly lower compared with those in the low YWHAE expression group. This result followed the same trend when comparing patients with early or late FIGO staging (*P* < 0.05) (Fig. 2d). Univariate and multivariate Cox regression analysis of YWHAE expression with age, pathological type, degree of differentiation, FIGO stage, and lymph node metastasis, demonstrated that YWHAE expression and FIGO staging are risk factors for the prognosis of epithelial ovarian malignancies (Fig. 2e).

#### YWHAE and HE4 expressions are related in ovarian cancer tissues

Next, the co-expression of YWHAE and HE4 was evaluated in 80 cases of ovarian cancer. A total of 0, three, nine, and 68 cases were YWHAE−/HE4−, YWHAE−/HE4+, YWHAE+/HE4−, and YWHAE+/HE4+, respectively. Spearman’s correlation analysis confirmed that YWHAE and HE4 expression is positively correlated in ovarian cancer (correlation coefficient Rs = 0.277, *P* = 0.013) (Table 3, Fig. 2f). Linear regression analysis showed that YWHAE and HE4 can influence the expression of each other (*P* < 0.05), and that the late FIGO stages are important factors affecting HE4 expression (Table 4).

**Table 3:** Correlation between YWHAE and HE4 in ovarian cancer

YWHAE	HE4		Cases
	−	+	
**−**	0	3	3
**+**	9	68	77
**Cases**	9	71	80

Sperman correlation coefficient Rs = 0.277, *P* = 0.013

**Table 4:** Linear regression analysis of YWHAE and HE4

	YWHAE score				HE4 score			
	Univariate		Multivariate		Univariate		Multivariate	
	β	*P*	β	*P*	β	*P*	β	*P*
HE4 score	0.307	0.009	0.307	0.009^a^				
YWHAE score					0.272	0.009	0.235	0.025^b^
Age at diagnosis	− 0.238	0.772			0.137	0.859		
FIGO stage	1.429	0.087			1.772	0.023	1.436	0.062
Differentiation	− 0.203	0.809			0.139	0.861		
Lymphatic metastasis	– 0.017	0.986			1.383	0.118		

^a^Represents multi-factor linear regression analysis, with YWHAE score as a variable, HE4 score as an argument;

^b^Represents multi-factor linear regression analysis, with HE4 score as a variable, with YWHAE score and FIGO stage as arguments

#### YWHAE expression correlates with ovarian cancer tissues

The Oncomine database showed that YWHAE was highly expressed in the cancer group compared to the normal tissue group. For ovarian cancer, YWHAE expression was significant in 185 ovarian carcinoma tissues compared with 10 ovarian surface epithelium tissues (Fig. 2g, h).

### YWHAE promotes ovarian cancer cell invasion, migration, and epithelial–mesenchymal transition potential

**Fig. 3:**
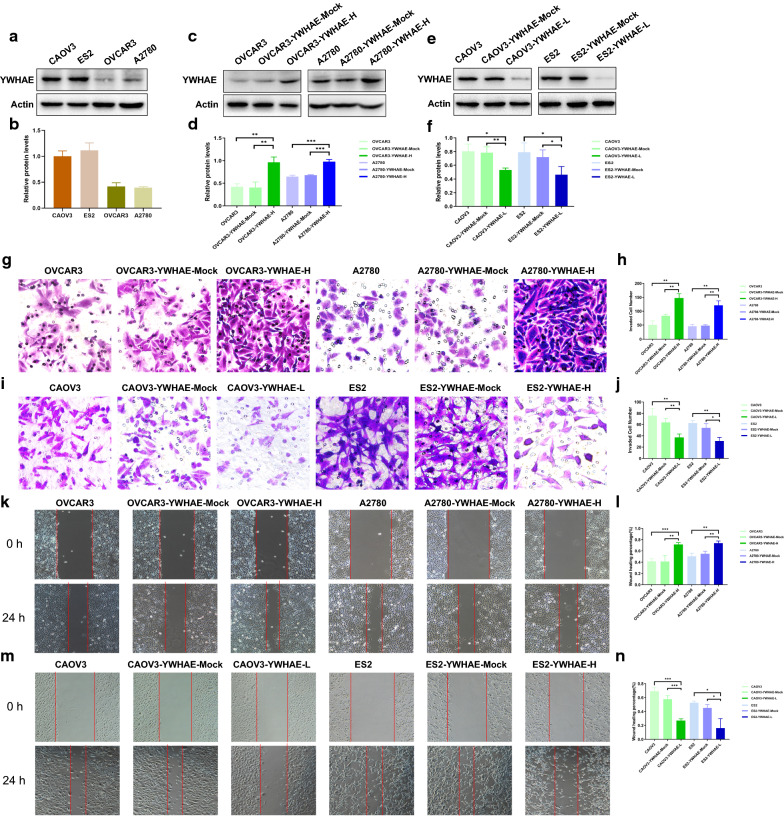
YWHAE affects cell invasion, migration, and epithelial–mesenchymal transition in ovarian cancer. **a**, **b** YWHAE expression in ovarian cancer cell lines. **c**, **d** Western blot analysis of YWHAE over-expression, mock-transduced, and untransduced OVCAR3 and A2780 cells. **e**, **f** Western blot of YWHAE knock-down, mock-transduced, and untransduced CAOV3 and ES2 cells. **g**, **h** Effects of high YWHAE expression on the invasion of ovarian cancer in OVCAR3 and A2780 cells. **i**, **j** Effects of low YWHAE expression on the invasion of ovarian cancer in CAOV3 and ES2 cells. **k**, **l** Effects of high YWHAE expression on the migration of ovarian cancer in OVCAR3 and A2780 cells. **m**, **n** Effects of low YWHAE expression on the migration of ovarian cancer in CAOV3 and ES2 cells

Analysis of YWHAE expression in several ovarian cancer cell lines revealed higher levels in CAOV3 and ES2 than in OVCAR3 and A2780 lines. Based on these results, CAOV3 and ES2 cells were used to establish YWHAE knockdown cell lines, whereas OVCAR3 and A2780 cells were used to establish stable YWHAE overexpression cell lines (Fig. 3a–f).

**Fig. 4:**
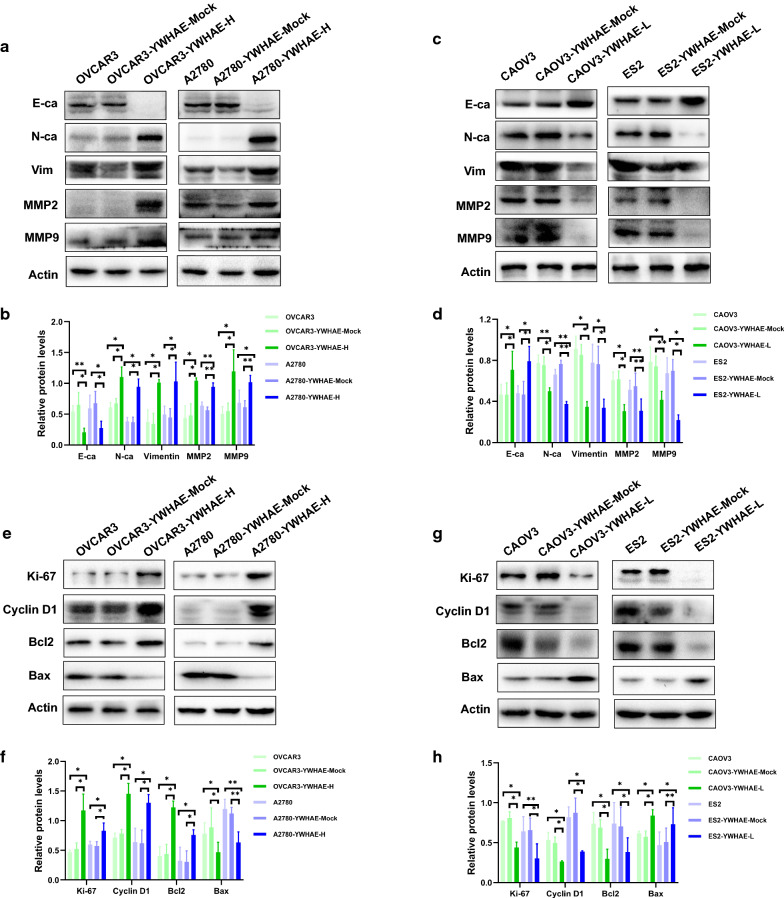
Western blot analysis of related proteins in ovarian cancer cells. **a**–**d** Expression of E-cadherin, N-cadherin, vimentin, MMP2, and MMP9 in ovarian cancer cells with high and low YWHAE expression, respectively. **e**–**h** Expression of Ki67, cyclin D, Bcl-2, and Bax in ovarian cancer cells with high and low YWHAE expression, respectively

The effect on ovarian cancer cell invasion and migration upon transient knockdown, or stable overexpression of YWHAE was next evaluated by transwell, and scratch experiments. Overall, the data revealed that both OVCAR3 and A2780 cells overexpressing YWHAE had significantly enhanced invasion and migration capacity in comparison with mock-transduced, and untransduced cells. In contrast, CAOV3 and ES2 cells reducing YWHAE showed weaker invasion and migration abilities compared with control cells (all *P* < 0.05) (Fig. 3g–n). To further explore the impact of YWHAE on the behaviour of ovarian cancer cells, the expression of epithelial and mesenchymal markers was evaluated in these cells by western blot. YWHAE-overexpression was found to be associated with higher levels of N-cadherin, vimentin, MMP2, and MMP9 (cell mesenchymal markers), while the epithelial marker E-cadherin was reduced when compared with cells in the control groups. The opposite trend was observed when YWHAE was knocked-down (both *P* < 0.05), with higher levels of E-cadherin and reduced levels of N-cadherin, vimentin, MMP2, and MMP9 (Fig. 4a–d). Altogether, these results demonstrate that YWHAE promotes invasion, migration, and epithelial–mesenchymal transition of epithelial ovarian cancer cells.

### YWHAE promotes ovarian cancer by inducing cell proliferation and cell cycle progression and inhibiting apoptosis

**Fig. 5:**
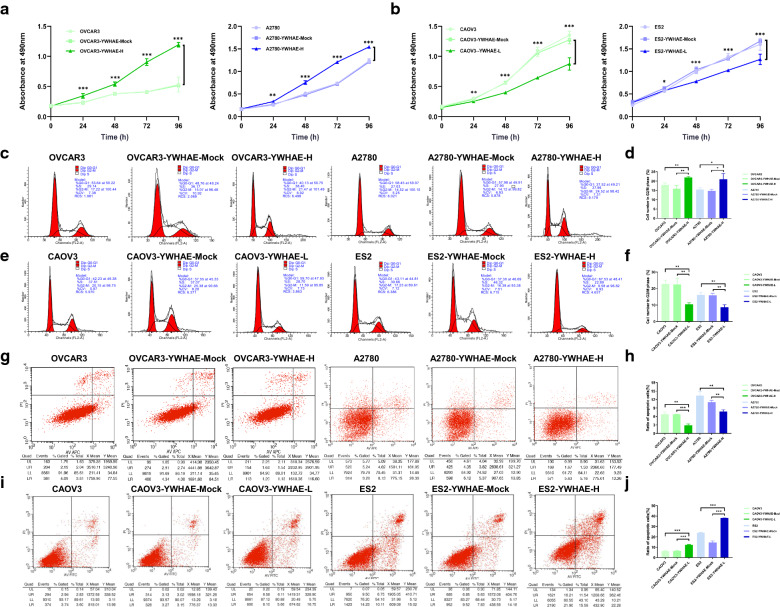
Effect of YWHAE on proliferation, apoptosis, and cell cycle of ovarian cancer cells. **a** YWHAE overexpression promoted the proliferation of OVCAR3 and A2780 ovarian cancer cells, as determined via MTT assay; **b** YWHAE knockdown inhibited the proliferation of CAOV3 and ES2 ovarian cancer cells, as determined via MTT assay. **c**, **d** Ovarian cells passed into G2/M phases following YWHAE overexpression; **e**, **f** G0/G1 phase arrested following YWHAE siRNA transfection; **g**, **h** YWHAE overexpression decreased apoptosis of OVCAR3 and A2780 cells; **i**, **j** YWHAE knockdown increased apoptosis of CAOV3 and ES2 cells

Additional assessment of the OVCAR3 and A2780 YWHAE overexpression cell lines further showed that most cells were in G2/M phase, suggesting that they were actively proliferating compared with mock-transduced and untransduced cells (all *P* < 0.05). Furthermore, these cells showed higher expression of Ki67, cyclin D1, and Bcl-2, and reduced levels of Bax (all *P* < 0.05). CAOV3 and ES2 knockdown YWHAE lines showed the opposite results, with a significantly low proportion of cells in the G2/M phase, reduced levels of proliferation markers, and increased levels of the Bax apoptosis marker. Furthermore, flow cytometry revealed that compared with the control group, apoptosis of OVCAR3 and A2780 cells was significantly reduced following YWHAE overexpression (*P* < 0.05). In contrast, apoptosis was significantly increased in OVCAR3 and A2780 cells when YWHAE expression was inhibited, compared with the control group (*P* < 0.05) (Figs. 4e–h, 5). Taken together, these results demonstrate that YWHAE expression can enhance the proliferation of ovarian cancer cells while also promoting cell cycle progression and inhibiting cellular apoptosis.

### Effect of YWHAE on the in vivo tumorigenesis of ovarian cancer cells

**Fig. 6:**
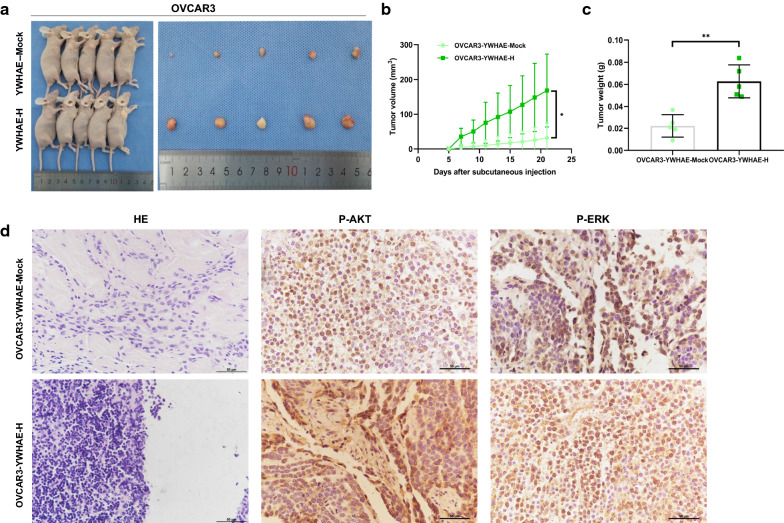
Impact of YWHAE on tumour formation and proliferation in vivo*.* **a** Subcutaneous xenografts of nude mice using OVCAR3 cells stably overexpressing YWHAE; **b**, **c** volume and quality changes of tumours; **d** haematoxylin & eosin staining and immunohistochemical staining of p-AKT and p-ERK in YWHAE-overexpressed and control tumours

To explore the effect of YWHAE on the tumorigenic ability of ovarian cancer cells, OVCAR3 cells overexpressing YWHAE, or a mock control, were injected into athymic nude mice. Assessment of the tumours formed 21 days after the cells were injected, revealed that tumours overexpressing YWHAE were significantly bigger, and weighed approximately 2.83 times more than the tumours observed in the control group. Moreover, the growth rate of the tumours produced by YWHAE-overexpressing cells was significantly faster compared with those in the control group (Fig. 6a–c). Immunohistochemistry of tumour biopsies revealed that AKT and ERK (p-AKT and p-ERK, respectively) expression was higher in OVCAR3 cells overexpressing YWHAE, further suggesting that YWHAE can promote the proliferation of tumour cells in vivo (Fig. 6d).

### YWHAE-induced cellular effects are mediated by the PI3K/AKT and MAPK signalling pathways

**Fig. 7:**
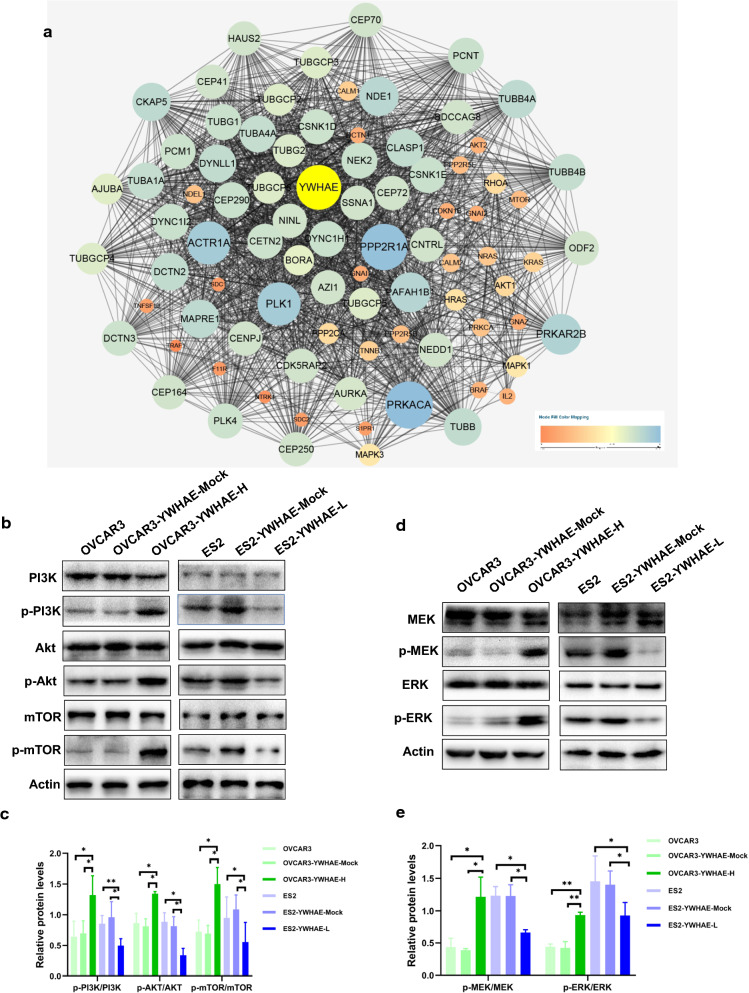
YWHAE induced cellular effects by PI3K/AKT and MAPK signalling pathways. **a** STRING database predicted the relevant molecules of YWHAE by PPI. **b**, **c** Expression of p-PI3K, PI3K, p-AKT, AKT, mTOR, and p-m-TOR in ovarian cancer cells with a high or low expression of YWHAE. **d**, **e** Expression of p-ERK, ERK, p-MEK, and MEK in ovarian cancer cells with a high or low expression of YWHAE

We used the STRING database to predict relevant YWHAE PPI and showed that YWHAE had direct or indirect interactions with PI3K, MAPK, ACTR1A, and cAMP-dependent protein kinase (Fig. 7a).

For a more detailed perspective on the underlying mechanisms triggered by YWHAE, the levels of several critical signalling molecules were evaluated by western blotting. The results showed that the ratio of p-PI3K/PI3K, p-AKT/AKT, p-m-TOR/mTOR, p-ERK/ERK, and p-MEK/MEK increased significantly in the presence of YWHAE overexpression, but were reduced upon YWHAE knockdown (all *P* < 0.05). The above results demonstrate that YWHAE can activate PI3K/AKT and MAPK signalling pathways (Fig. 7b–e).

**Fig. 8:**
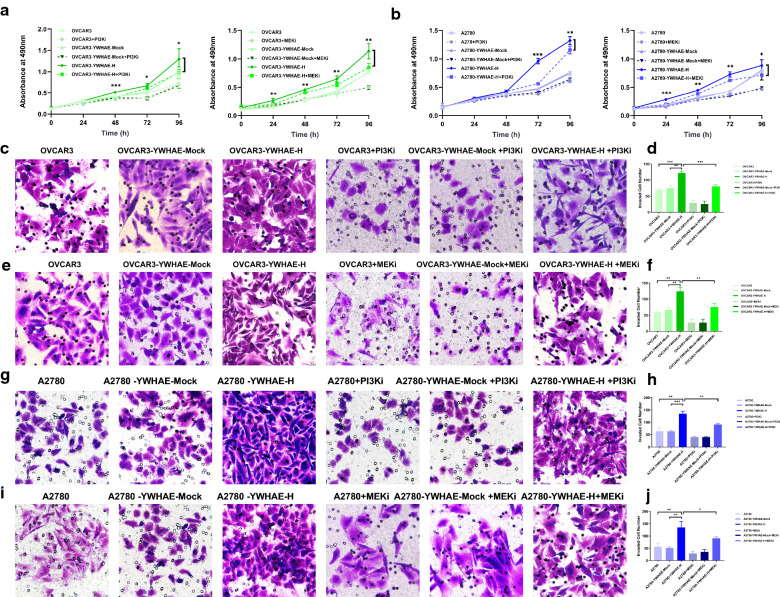
PI3K and MEK inhibitors reduce MTT and invasion capabilities of ovarian cancer cells. **a**, **b** PI3K and MEK inhibitors reduced the proliferation capability of YWHAE over-expression, mock-transduced, and untransduced OVCAR3 and A2780 cells; **c**–**j** PI3K and MEK inhibitors reduce the invasion capability in YWHAE-high expression, mock-transduced, and untransduced OVCAR3 and A2780 cells

**Fig. 9:**
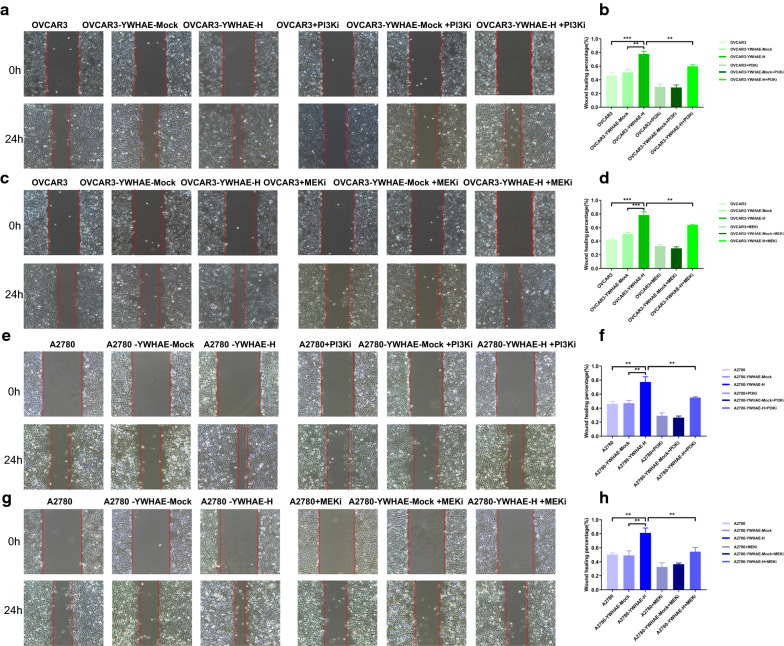
PI3K and MEK inhibitors reduce the ability of ovarian cancer cells to migrate

To further explore the role of these two signalling pathways on YWHAE-induced cellular effects, PI3K (GDC-0941) or MEK (PD98059) inhibitors were used. The invasion and migration experiments were repeated in the presence of these inhibitors, revealing that blockage of both PI3K/AKT and MAPK signals significantly weakened the pro-invasion and pro-migration effect promoted by YWHAE overexpression (*P* < 0.05). Moreover, we showed that the proliferation of OVCAR3 cells overexpressing YWHAE was significantly reduced in the presence of GDC-0941 or PD98059 (*P* < 0.05) (Figs. 8, 9).

The above results demonstrate that YWHAE can impact the invasion, migration, and proliferation potentials of ovarian cancer cells, as well as other malignant biological behaviours, through the PI3K/AKT and the MAPK signalling pathways.

## Discussion

Ovarian cancer, a tumour of the female reproductive system, has a high mortality rate. Although a variety of targeted drugs for ovarian cancer are used in the clinical setting, the high mortality rate still represents a serious threat to the lives and health of women worldwide.

The YWHAE protein is widely expressed in eukaryotes, having been detected in wheat [19], giant trematodes in goat blood cells [20], liver flukes [21], and mosquitoes [22]. Moreover, YWHAE has been identified as an important element in human retinal photoreceptor rod cells [23], and is involved in the differentiation of adipose-derived mesenchymal stem cells into osteoblasts, thus enhancing the body’s osteogenic ability [24]. In contrast, some studies have detected a peak in YWHAE expression 168 h following partial liver resection, preventing cell cycle progression and negatively regulating liver regeneration [25]. These discordant findings therefore suggest that YWHAE may have a two-way regulatory effect on the cell cycle.

As YWHAE was originally identified in the brain, its pathological effects were initially investigated in the field of neurological diseases, such as Parkinson’s [26] and Alzheimer’s disease [27], brain excitotoxic injury [28], and myocardial ischemia reperfusion [29], among others. These studies agreed that the mechanism by which YWHAE could impact nerve cells may be related to mitochondrial dysfunction and apoptosis regulation.

In recent years, studies have suggested that abnormal expression of YWHAE may also play an important role in the occurrence and development of tumours. Liang et al. found that YWHAE is highly expressed in kidney cancer tissues, and in vitro experiments demonstrated that YWHAE promotes abnormal proliferation of tumour cells [30]. In gastric cancer cell lines, YWHAE expression is significantly upregulated, and can inhibit cell proliferation, invasion, and migration by reducing the expression of MYC and CDC25B; whereas MYC induces cell proliferation, invasion, and migration by enhancing CDC25B, and reducing YWHAE expression [12, 31]. In breast cancer, YWHAE expression is related to tumour size, lymph node metastasis, and patient prognosis, as well as breast cancer resistance to chemotherapy. Indeed, YWHAE overexpression significantly increases breast cancer cell proliferation, migration, and invasion, whereas reduced YWHAE expression prevents Snail and Twist expression in breast cancer cells [32]. Although high expression of YWHAE has been described in colorectal, liver, kidney, breast, gastric, and oesophageal cancers, its specific mechanism of action remains unclear.

Among the malignant tumours of the female reproductive system, YWHAE is more commonly reported upon genetic testing of uterine sarcoma cells. Endometrial stromal sarcoma carrying the YWHAE–NUTM2 (or YWHAE–FAM22) fusion gene has obvious malignant biological effects, such as enhanced invasion and drug resistance. Additionally, the prognosis of patients harbouring such genetic abnormality is poor [33, 34]. Sylvain et al. [35] performed gene chip detection on matched tumour samples from six patients with advanced high-grade epithelial ovarian cancer before and after chemotherapy. The results showed that 54 genes that recurred after chemotherapy were downregulated, whereas 121 genes, including *YWHAE*, were upregulated. This change in the expression profile suggests that YWHAE may be related to ovarian cancer invasion, proliferation, and drug resistance. Sun et al. used the Gene Expression Omnibus database to analyse the relationship between ovarian cancer and diabetes, and found that 10 key genes, including *YWHAE*, are important links in the regulation of redox reactions, and carboxylic acid metabolism in the body [36]. Based on these results, they believe that ovarian cancer is related to sugar metabolism, and that certain key metabolism-related genes and proteins could be used as potential targets for the treatment of ovarian cancer.

Taken together, the results described in the present study demonstrate that YWHAE and HE4 are interacting proteins, and that YWHAE is significantly associated with advanced stage cancers and poor patient outcomes. This suggests that high YWHAE expression may represent a risk factor for the prognosis of ovarian cancer. Overall, YWHAE showed a similar cancer-promoting effect as observed for HE4, contributing to the occurrence and development of ovarian cancer.

Through induced differential expression of YWHAE and in vivo experiments, we also demonstrated that YWHAE contributes to ovarian cancer cell invasion, epithelial–mesenchymal transition, and migration, as well as to the enhanced proliferative and anti-apoptotic responses of these cells.

Previous studies have confirmed that HE4 plays an important role in the metastasis and adhesion of ovarian cancer cells, with low levels of HE4 preventing the activation of ERK and EGFR in ovarian cancer cells. Therefore, it is thought that HE4 may influence the biological behaviour of cancer cells in the ovaries through the EGFR and MAPK signalling pathways; however, the specific underlying mechanism is still unclear. Studies have reported that HE4 can affect the cell cycle (G0/G1 phase), as well as cellular migration and invasion capabilities by regulating ERK/MAPK and the expression of MMP9, MMP2, and cathepsin B [37]. In accordance with its role as an interaction protein of HE4, YWHAE was also shown to affect the malignant biological behaviour of ovarian cancer cells through the above-mentioned signalling pathways.

In breast cancer, YWHAEτ acts together with 1,3-DCQA (eicosanylquinic acid) to prevent the proliferation and metastasis of cancer cells through the Jak/PI3K/AKT and Raf/ERK pathways, and by inducing the Bad/Bax/caspase 9 apoptosis pathway [38]. YWHAEζ overexpression can regulate the expression of the protein Snail by activating the PI3K/AKT pathway, thereby promoting the proliferation, migration, and invasion of glioma cells. This protein therefore represents a potential prognostic marker and therapeutic target for glioma [39]. In colorectal cancer, YWHAEσ acts as a tumour suppressor gene. However, COPS5 and LASP1, through PI3K/AKT-dependent signalling, stimulate YWHAEσ ubiquitination and degradation. This removes the tumour suppressor activity of YWHAEσ that promotes the progression of colorectal cancer [40].

Relevant studies have shown that YWHAE can inhibit cell apoptosis in HT-29 colorectal cancer cells [41], and that this process can be reversed by non-steroidal anti-inflammatory drugs. The inhibition of apoptosis may be related to the ability of YWHAE to interfere with mitochondrial pro-apoptotic mechanisms, and the activation of transcription factors FKHRL1 and Bad [42]. Moreover, 4-amino-2-trifluoromethyl-phenyl retinoic acid can induce G0/G1 phase arrest in SGC-7901 gastric cancer cells by downregulating YWHAE [43]. We found that PI3K/AKT pathway node proteins (PI3K, AKT, and mTOR), and MAPK pathway node proteins (MEK and ERK) were significantly upregulated in ovarian cancer cells overexpressing YWHAE. Importantly, inhibition of these pathways prevented the pro-invasion, pro-migration, and pro-proliferation effects induced by YWHAE. Therefore, our results suggest that YWHAE promotes the malignant biological behaviour of epithelial ovarian cancer through activation of the PI3K/AKT and MAPK pathways.

## Conclusions

This study described, for the first time, that YWHAE and HE4 interact and co-localise in cells. We demonstrated that YWHAE expression was significantly increased in ovarian cancer tissues, which was a risk factor for the prognosis of ovarian cancer. Moreover, we showed for the first time that YWHAE could promote the invasion, migration, and proliferation of epithelial ovarian cancer through PI3K/AKT and MAPK pathways. Our results suggest that YWHAE could be used as a prognostic factor and may trigger new research ideas for further understanding the underlying pathogenesis and improving the diagnosis and treatment of ovarian cancer.

## Supplementary Information

**Additional file 1.** Verification of three HE4-siRNAs transfection.

## Data Availability

Not applicable.

## Abbreviations

HE4: Human epididymis protein 4
FIGO: International Union of Obstetrics and Gynaecology
GSEA: Gene Set Enrichment Analysis
FBS: Foetal bovine serum
DMSO: Dimethyl sulfoxide
FITC: Fluorescein isothiocyanate
DAPI: 4′,6-Diamidino-2-phenylindole
PBS: Phosphate-buffered saline
PVDF: Polyvinylidene fluoride membrane
KEGG: Kyoto Encyclopedia of Genes and Genomes
GO: Gene Ontology
