# Supplementary figures and images for: YWHAE as an HE4 interacting protein can influence the malignant behaviour of ovarian cancer by regulating the PI3K/AKT and MAPK pathways
Source: Cancer Cell Int. 2021 Jun 9;21:302. doi: 10.1186/s12935-021-01989-7 (PMC8190858; doi:10.1186/s12935-021-01989-7)

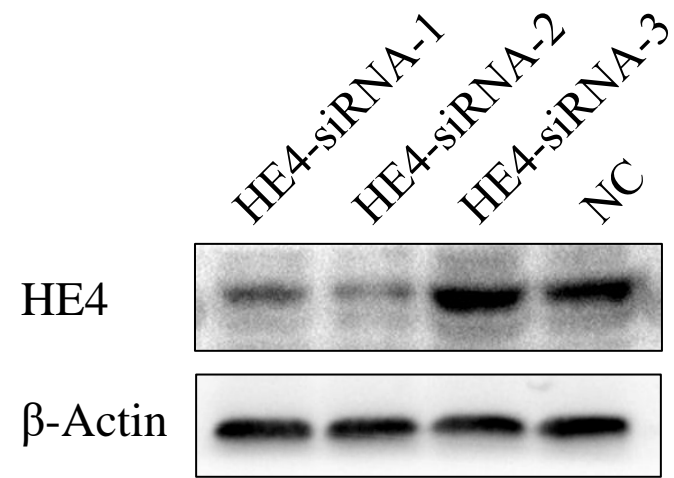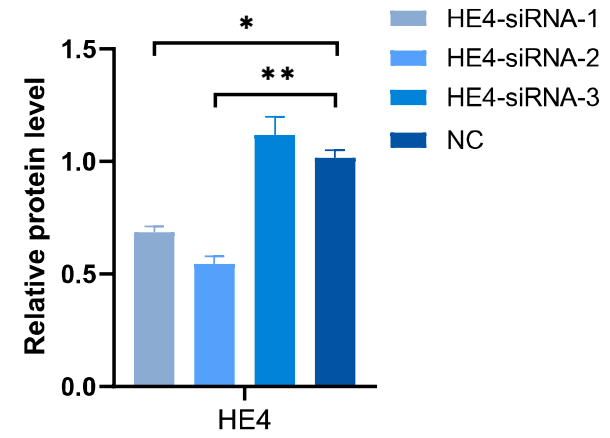

Supplement: Supplementary file 1 — Additional file 1. Verification of three HE4-siRNAs transfection. [file 12935_2021_1989_MOESM1_ESM.pdf]
